# Urolithin A protects severe acute pancreatitis‐associated acute cardiac injury by regulating mitochondrial fatty acid oxidative metabolism in cardiomyocytes

**DOI:** 10.1002/mco2.459

**Published:** 2023-12-19

**Authors:** Yue Yang, Qian Hu, Hongxin Kang, Juan Li, Xianlin Zhao, Lv Zhu, Wenfu Tang, Meihua Wan

**Affiliations:** ^1^ Department of Integrated Traditional Chinese and Western Medicine West China Hospital of Sichuan University Chengdu China; ^2^ Digestive Department The First People's Hospital of Shuangliu District Chengdu China

**Keywords:** acute cardiac injury, fatty acid metabolism, mitochondrial injury, severe acute pancreatitis, Urolithin A

## Abstract

Severe acute pancreatitis (SAP) often develops into acute cardiac injury (ACI), contributing to the high mortality of SAP. Urolithin A (UA; 3,8‐dihydroxy‐6H‐dibenzopyran‐6‐one), a natural polyphenolic compound, has been extensively studied and shown to possess significant anti‐inflammatory effects. Nevertheless, the specific effects of UA in SAP‐associated acute cardiac injury (SACI) have not been definitively elucidated. Here, we investigated the therapeutic role and mechanisms of UA in SACI using transcriptomics and untargeted metabolomics analyses in a mouse model of SACI and in vitro studies. SACI resulted in severely damaged pancreatic and cardiac tissues with myocardial mitochondrial dysfunction and mitochondrial metabolism disorders. UA significantly reduced the levels of lipase, amylase and inflammatory factors, attenuated pathological damage to pancreatic and cardiac tissues, and reduced myocardial cell apoptosis and oxidative stress in SACI. Moreover, UA increased mitochondrial membrane potential and adenosine triphosphate production and restored mitochondrial metabolism, but the efficacy of UA was weakened by the inhibition of CPT1. Therefore, UA can attenuate cardiac mitochondrial dysfunction and reduce myocardial apoptosis by restoring the balance of mitochondrial fatty acid oxidation metabolism. CPT1 may be a potential target. This study has substantial implications for advancing our understanding of the pathogenesis and drug development of SACI.

## Graphical abstract

Potential mechanisms underlying the role of Urolithin A in improving inflammatory injury and mitochondrial dysfunction in SACI. UA regulates CD36 and CPT1 to balance mitochondrial fatty acid oxidative metabolism and attenuate mitochondrial dysfunction through activation of the PPARα/PGC1 signaling pathway, as well as reduces apoptosis and ultimately attenuates myocardial inflammatory damage.

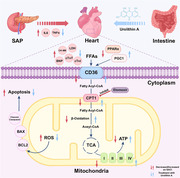

## 1 INTRODUCTION

Severe acute pancreatitis (SAP) is an inflammatory response in the pancreas itself and surrounding tissues due to various causes, such as gallstones, and has a mortality rate of up to 30%.^1^, ^2^ Complications of severe myocardial injury and dysfunction are prevalent in cases of SAP, substantially amplifying the potential for morbidity and mortality.^3^ Several studies have provided empirical evidence supporting the development of myocardial dysfunction in patients with SAP. Commonly observed manifestations include arrhythmias, cardiogenic shock, and myocarditis.^4^, ^5^, ^6^ The pathogenesis of SAP‐associated acute cardiac injury (SACI) is notably intricate, and a comprehensive understanding of its pathogenesis is still insufficient. The current understanding of the pathophysiological mechanism of SACI draws parallels with sepsis‐induced cardiomyopathy (SICM), indicating that the structural damage and dysfunction of the heart are linked to heightened levels of risk factors such as trypsin, inflammation‐related factors, reactive oxygen species (ROS), and endotoxins.^7^, ^8^, ^9^ Treatment for SACI mainly consists of supportive care measures, as there are no specific pharmacological interventions available. Consequently, the mortality rate of SACI remains high. Given the importance of this matter, it is essential to investigate the potential pathogenesis of myocardial injury associated with acute pancreatitis and pursue studies aimed at discovering potentially efficacious therapeutic agents.

The normal heart generates most of its adenosine triphosphate (ATP) through oxidative phosphorylation in the mitochondria, of which approximately 60−90% of the energy is derived from mitochondrial fatty acid oxidation (FAO).^10^ However, when the heart is ischemic and hypoxic, FAO is inhibited, and a large amount of activated long‐chain fatty acids accumulate in the cells and mitochondrial membranes. This causes lipid accumulation, disrupts mitochondrial function and triggers apoptosis, which eventually leads to cardiac damage.^11^, ^12^, ^13^ These effects may be attributed to the dysregulation of key molecular mechanisms engaged in fatty acid metabolism, such as fatty acid transporter protein cluster of differentiation 36 (CD36), carnitine palmitoyl‐transferase 1 (CPT1), PPAR‐γ coactivator‐1‐α (PGC1‐α), and peroxisome proliferator‐activated receptor‐α (PPAR‐α).^14^, ^15^, ^16^, ^17^ Considering the limited knowledge surrounding the mechanisms of fatty acid metabolism in SACI, a promising approach for treating this condition could involve targeting FAO‐related factors and modulating mitochondrial fatty acid energy metabolism.

Urolithin A (UA; 3,8‐dihydroxy‐6H‐dibenzopyran‐6‐one) is a polyphenolic compound that is naturally synthesized by the metabolism of the gut microbiota through the consumption of foods rich in ellagitannins and ellagic acid, such as pomegranate, berries, walnuts and strawberries.^18^, ^19^, ^20^, ^21^ The therapeutic efficacy of UA has been substantiated in multiple studies across a range of diseases, such as central nervous system disorders, cardiovascular disease, and metabolic diseases. This efficacy can be attributed to its biological properties, including anti‐inflammatory and antioxidant properties, maintenance of mitochondrial function, and regulation of metabolic homeostasis.^22^ Studies conducted using a model of pancreatic β‐cells induced by glycolipotoxicity have suggested that UA promotes mitochondrial autophagy, mitigates endoplasmic reticulum stress, and suppresses the TXNIP/NLRP1/IL‐1β inflammatory signaling pathway.^23^ Similarly, UA has emerged as a promising compound in cardiovascular studies. It reveals favorable outcomes by reducing serum lactate dehydrogenase (LDH) and creatine kinase MB isoenzyme (CK‐MB) levels, decreasing myocardial apoptotic cells, and diminishing infarct size in mice undergoing ischemia‒reperfusion injury.^24^ Moreover, UA mediates cholesterol transport, which reduces plasma lipid levels and atherosclerotic plaques.^25^ Apart from this, it boasts the capability to attenuate cardiac dysfunction in a rat model of diabetic cardiomyopathy.^26^ In addition, UA exhibits notable effects in attenuating triglyceride (TG) accumulation and boosting FAO.^27^, ^28^ Nonetheless, the effectiveness of UA in the treatment of SACI and its specific mechanism have yet to be fully elucidated.

Therefore, the core purpose of this research was to investigate the potential cardioprotective effect of UA in a SACI mouse model and to assess its impact on primary mouse cardiomyocytes. Mitochondrial function and energy metabolism were assessed to explore the underlying mechanisms of UA‐mediated cardiomyocyte protection against SACI.

## 2 RESULTS

### 2.1 UA has an effective therapeutic effect on SACI

**FIGURE 1:**
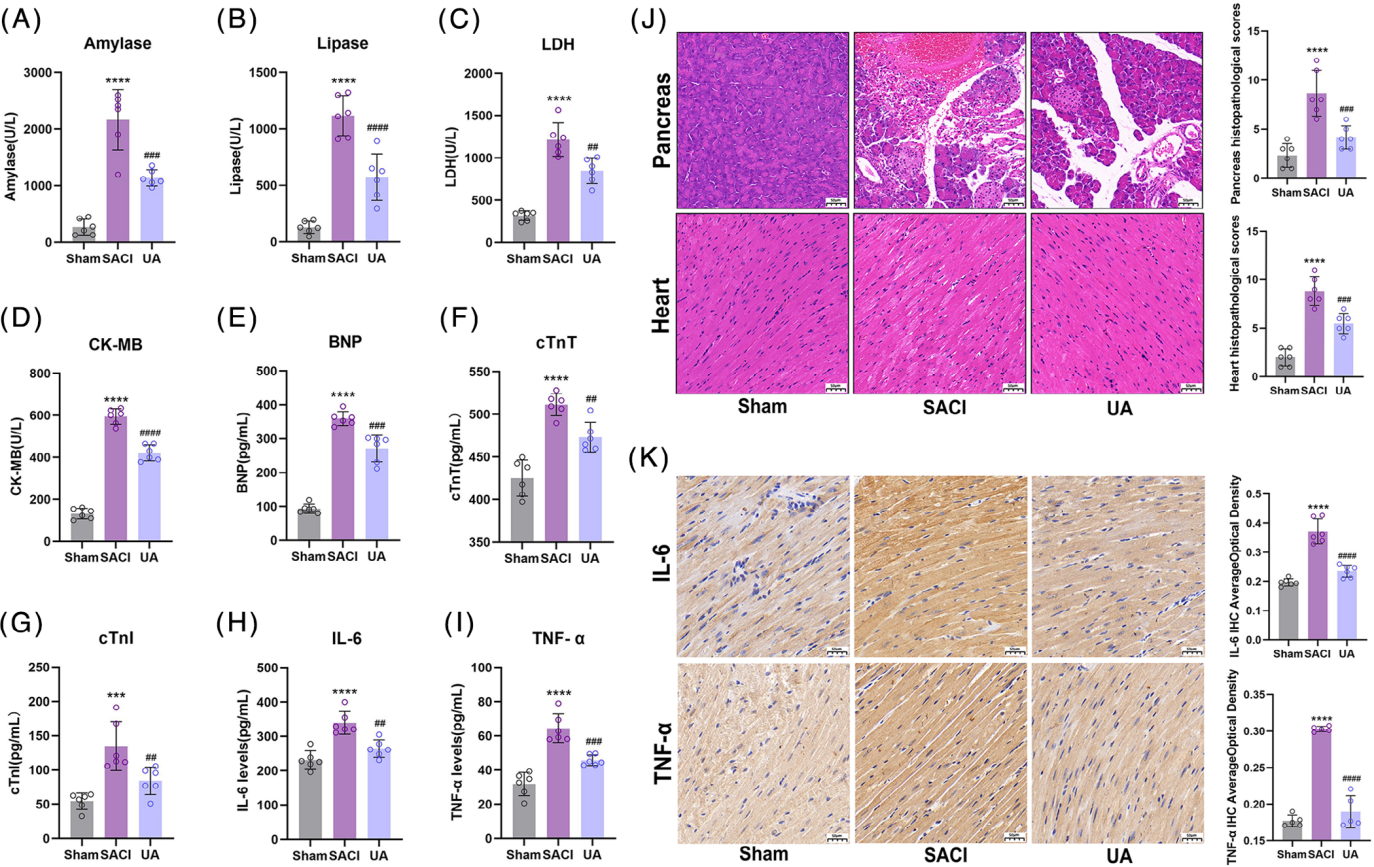
Urolithin A has effective therapeutic effect on SACI. (A and B) Serum amylase and lipase levels in the sham group (sham), severe acute pancreatitis‐associated acute cardiac injury group (SACI), and SACI plus Urolithin A treatment group (UA) (*n* = 6). (C–G) Serum levels of LDH, CK‐MB, BNP, cTnT, cTnI in mice from sham, SACI, and UA groups (*n* = 6). (H and I) Serum levels of IL‐6 and TNF‐α in mice from sham, SACI, and UA groups (*n* = 6). (J) Pathological images and histopathological scoring of H&E staining on pancreatic and cardiac tissues (scale bar = 50 μm; *n* = 6). (K) IL‐6 and TNF‐α expression in the heart tissues of mice as detected by immunohistochemistry (scale bar = 50 μm; *n* = 6). The results are presented as the mean ± SD. ****p < 0.0001 vs. sham group; ##*p* < 0.01, ###*p* < 0.005, ####*p* < 0.0001 vs. SACI group, by one‐way ANOVA tests followed by Tukey tests.

The data illustrated in Figures 1A, B, and J demonstrated a substantial increase in serum amylase and lipase levels and pancreatic pathological scores in the SACI group compared with the sham group.^29^ Moreover, cardiac injury was confirmed by increases in the serum levels of cardiac injury indicators such as LDH, CK‐MB, brain natriuretic peptide (BNP), cardiac troponin T (cTnT), cardiac troponin I (cTnI), and cardiac pathology scores in the SACI group compared with those in the sham group (Figures 1C–G). These results indicated that the animal models meet the diagnostic criterion for SACI.

Based on our studies (Figures S1), we discovered that intraperitoneal treatment with UA 1 h before and 3 h after modeling was optimal for SACI mice, which served as the basis for the subsequent experiments of UA treatment points.

UA significantly decreased the serum amylase, lipase, LDH, CK‐MB, BNP, cTnT, cTnI levels and lowered pancreatic and cardiac histopathology scores in SACI group (Figures 1A–G and J). Additionally, UA inhibited the increased proinflammatory factor interleukin‐6 (IL‐6) and tumor necrosis factor‐α (TNF‐α) levels in the serum and heart tissues of SACI mice (Figures 1H, I, and K). These outcomes indicate that UA could alleviate inflammatory injury in the pancreas and heart of SACI mice.

### 2.2 Effect of UA on mitochondria‐related genes in SACI mice

**FIGURE 2:**
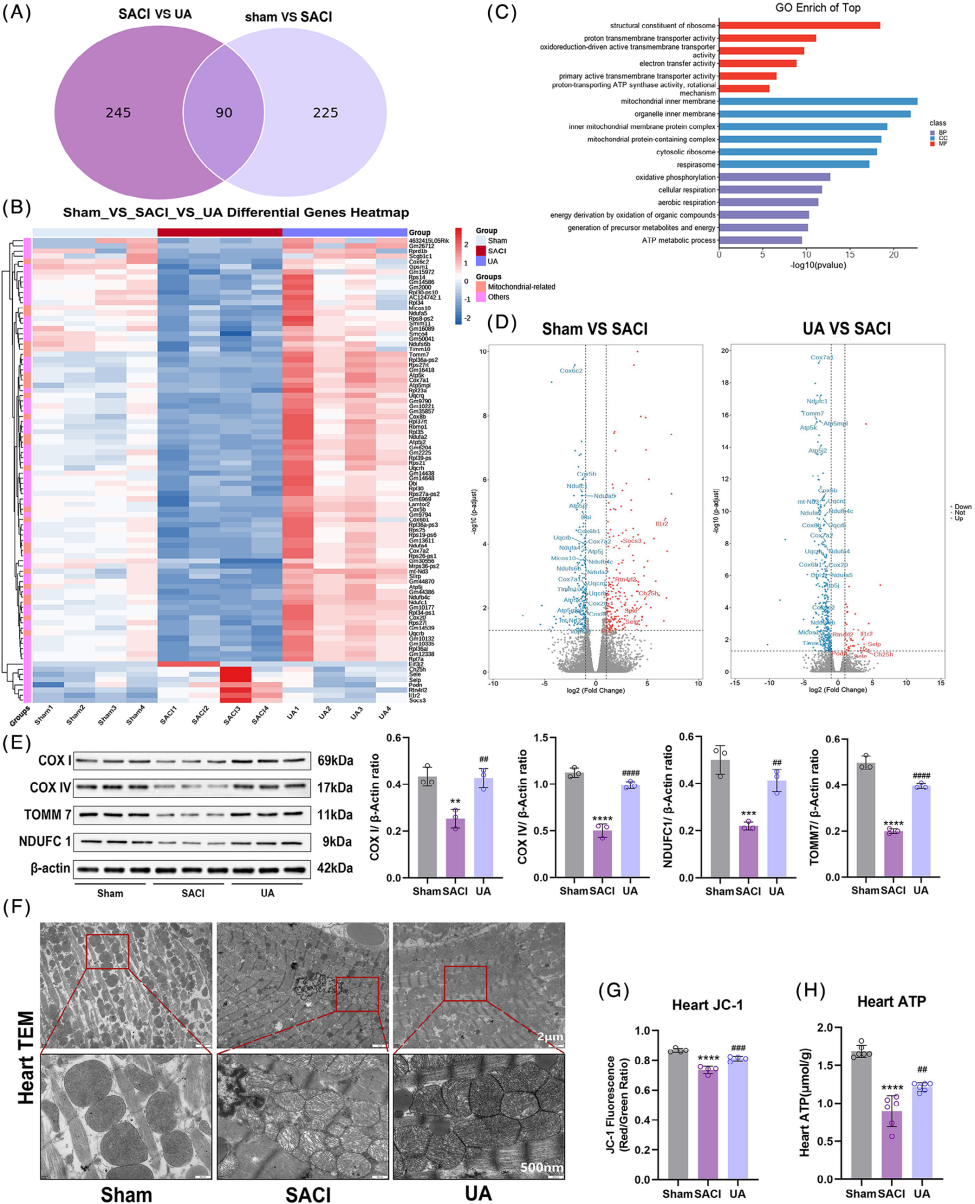
Effect of Urolithin A on mitochondria‐related genes in SACI mice. (A) Venn diagram of gene expression in sham group vs. SACI group and SACI group vs. UA group. (B) Heatmap analysis of 90 overlapping gene expressions in three groups (*n* = 4). (C) GO term analysis of 90 overlapping gene expressions in the SACI group vs. UA group. (D) Volcano plots of differential genes in the sham group vs. SACI group and UA group vs. SACI group. (E) Western blotting and quantitative analysis of COX I, COX IV, TOMM7, and NDUFC1 in cardiac tissues of the sham, SACI, and UA groups (*n* = 3). (F) Representative transmission electron microscopy (TEM) images of cardiac tissues in the sham, SACI, and UA groups (scale bar = 2 μm; 500 nm). (G) Mitochondrial membrane potential changes in isolated purified heart mitochondria in the sham, SACI, and UA groups. (JC‐1 fluorescence: red/green ratio; *n* = 4). (H) Changes in total ATP concentration in heart tissues in the sham, SACI, and UA groups (*n* = 6). The results are presented as the mean ± SD. **p < 0.01, ***p < 0.005, ****p < 0.0001 vs. sham group; ##*p* < 0.01, ###*p* < 0.005, ####*p* < 0.0001 vs. SACI group, by one‐way ANOVA tests followed by Tukey tests.

To reveal the potential mechanisms of UA for the treatment of SACI, RNA‐seq analysis was performed for mouse heart tissues. Differentially expressed genes (DEGs) were identified by applying the criteria of FPKM > 1 and an adjusted *p* value < 0.05. A total of 315 DEGs were detected between the SACI and sham groups, and 335 DEGs were detected between the SACI and UA groups. There were 90 overlapping DEGs shared among these groups, so it is reasonable to speculate that UA could exert its protective effects on SACI by regulating these 90 DEGs (Figure 2A). UA treatment resulted in a notable increase in the expression levels of mitochondria‐related genes among the 90 identified DEGs (Figure 2B). GO terminology analysis unveiled that the primary functional terms associated with these DEGs were the mitochondrial inner membrane and related complexes, electron transport activity, ATP synthase activity, and oxidative phosphorylation‐related energy metabolism (Figure 2C). Volcano analysis and Western blotting analysis provided compelling evidence of the decreased expression of translocase of outer mitochondrial membrane 7 (TOMM7) in SACI (Figures 2D and E), implying a disruption in the protein transport and stability machinery of mitochondria.^29^ Furthermore, DEGs involved in the mitochondrial respiratory electron transport chain (Ndufc1, Ndufa2, Ndufa4, Ndufa5, mt‐Nd3, and Uqcrq), which possesses a strong tie with the functioning of mitochondrial respiratory complex I‐IV, were downregulated in the SACI group (Figure 2D). UA upregulated their expression, restored COX I and COX IV activities engaged in the electron transfer chain (Figures 2D and E), and further restored myocardial mitochondrial membrane potential (Δ*Ψ*_m_) (Figure 2G). The expression levels of ATP synthases implicated in mitochondrial energy metabolism (Atp5mpl, Atp5k, Atp5j2, etc.) were decreased under the influence of SACI. However, UA effectively restored ATP synthase activity and increased the ATP production that was reduced by SACI (Figures 2D and H). Furthermore, transmission electron microscopy (TEM) revealed that UA increased the ability to repair structural damage in mitochondria within SAP‐influenced hearts, including mitochondrial swelling, disorganization, and partial dissolution and breakage of mitochondrial cristae (Figure 2F). To recapitulate, UA holds promise in reducing SACI damage by restoring mitochondrial morphological structure and ameliorating dysfunction in mitochondrial energy metabolism.

### 2.3 UA reduces myocardial apoptosis

**FIGURE 3:**
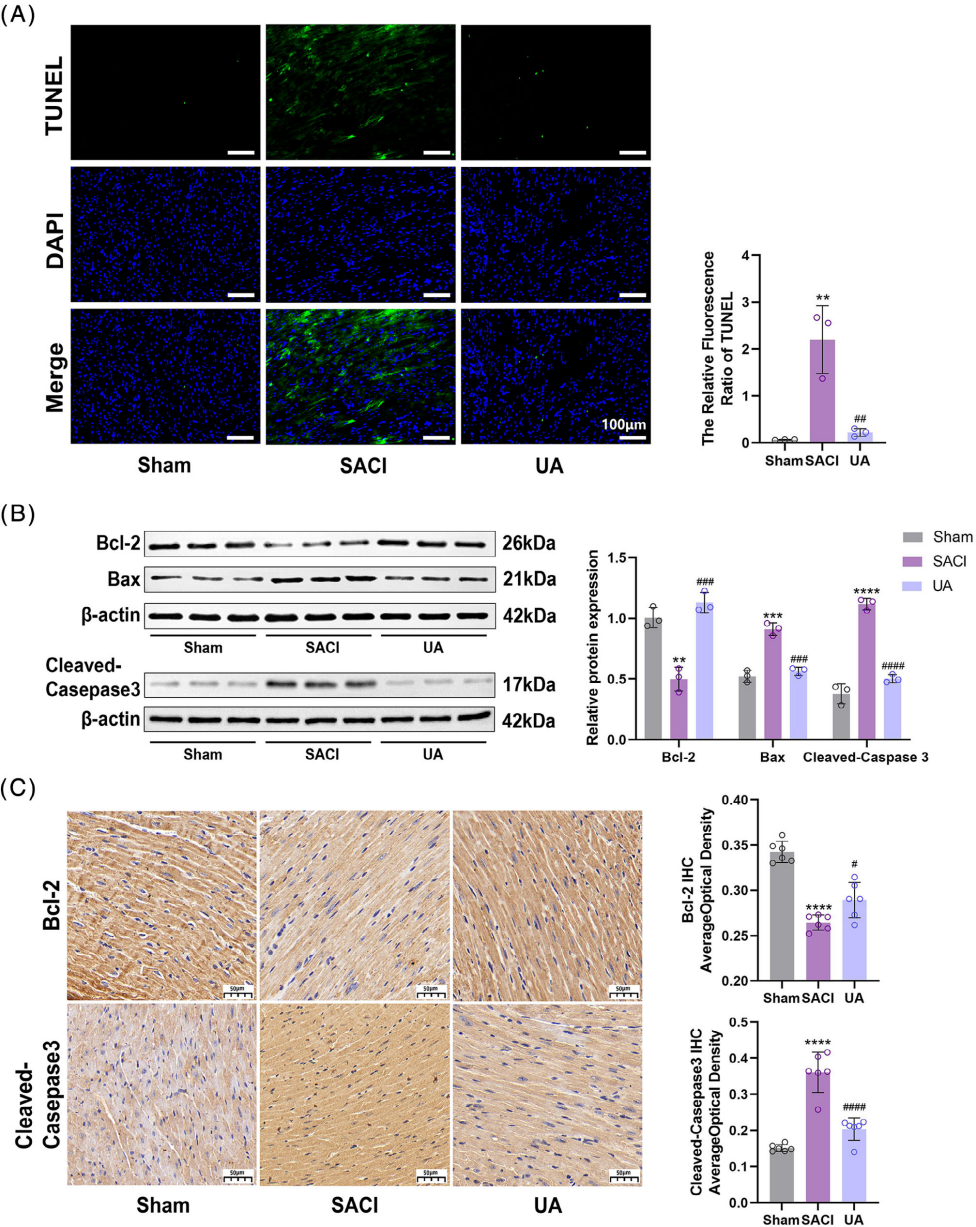
Urolithin A reduces myocardial apoptosis. (A) Representative TUNEL staining images of heart tissues in the sham, SACI and UA groups (scale bar = 100 μm; *n* = 3). (B) Western blotting and quantitative analyses of Bcl‐2, Bax, Cleaved‐Caspase3 in cardiac tissues of the sham, SACI, and UA groups (*n* = 3). (C) Bcl‐2 and Cleaved‐Caspase3 expression in the heart tissues of mice as detected by immunohistochemistry (scale bar = 50 μm; *n* = 6). The results are presented as the mean ± SD. **p < 0.01, ***p < 0.005, ****p < 0.0001 vs. sham group; #*p* < 0.05, ##*p* < 0.01, ###*p* < 0.005, ####*p* < 0.0001 vs. SACI group, by one‐way ANOVA tests followed by Tukey tests.

Cardiomyocyte mitochondria exhibit a propensity to initiate the apoptotic pathway under inflammation or oxidative stress.^30^ The findings depicted in Figure 3A indicate a substantial increase in the number of apoptosis‐positive cells within SACI hearts, and this effect was subsequently mitigated in the UA group. Western blotting and immunohistochemistry revealed that UA treatment upregulated the expression of the antiapoptotic protein Bcl‐2 while downregulating the expression of the proapoptotic proteins Bax and Cleaved‐Caspase3 (Figures 3B and C), which are key components of the mitochondrial death pathway. These changes strongly support the notion that UA protects cardiomyocytes against apoptosis by modulating the mitochondrial apoptosis pathway.

### 2.4 UA restores the energy metabolism disorder of SACI

**FIGURE 4:**
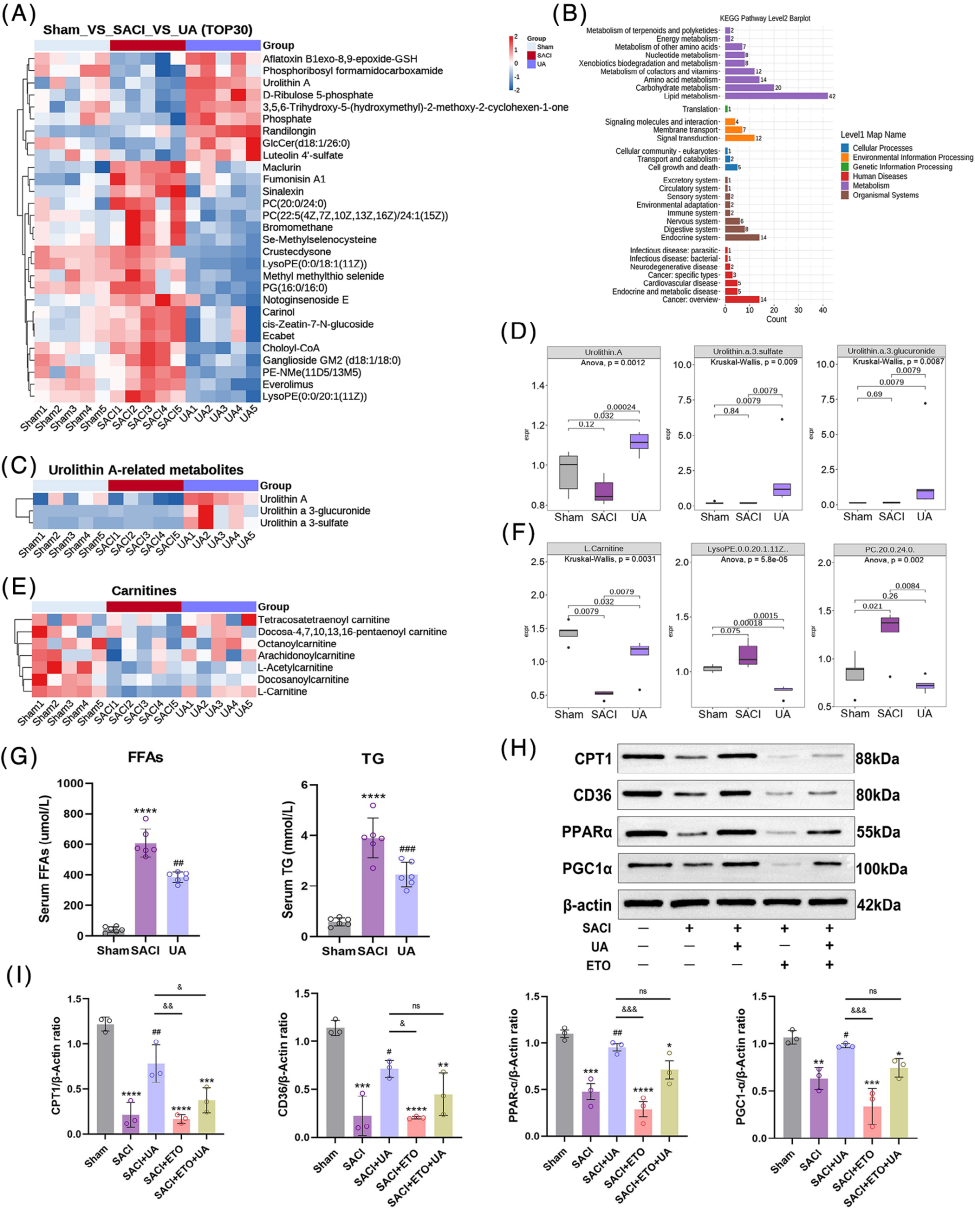
Urolithin A restores the energy metabolism disorder of SACI. (A) Heatmap analysis of top 30 differentially expressed metabolites in the sham, SACI, and UA groups (*n* = 5). (B) KEGG pathway analysis in the sham, SACI, and UA groups. (C and D) Urolithin A metabolites measured by untargeted metabolomics in the sham, SACI, and UA groups (*n* = 5). (E and F) Carnitines and glycerophospholipid metabolites measured by untargeted metabolomics in the in the sham, SACI, and UA groups (*n* = 5). (G) Serum Free fatty acid and triglyceride levels in the sham, SACI, and UA groups (*n* = 6). (H and I) Western blotting and quantitative analyses of CPT1, CD36, PPAR‐α, and PGC1‐α in cardiac tissues (*n* = 3). The results are presented as the mean ± SD. *p < 0.05, **p < 0.01, ***p < 0.005, ****p < 0.0001 vs. sham group; #*p* < 0.05, ##*p* < 0.01, ###*p* < 0.005 vs. SACI group, &*p* < 0.05, &&*p* < 0.01 vs. SACI+UA group, by one‐way ANOVA tests followed by Tukey tests. Kruskal–Wallis tests and Wilcox tests used in D and F.

To gain insights into the mechanisms by which UA regulates mitochondria under conditions of SACI, we adopted untargeted metabolomics for metabolite analysis. The heart sample data of the three groups were processed by adopting partial least squares discrimination analysis (PLS‐DA). The models' reliability was subsequently assessed through cross‐validation. The PLS‐DA model with VIP > = 1 (variable importance in the projection) was subsequently put into use and intergraded with independent samples *t*‐test (*p* < 0.05) to screen out differentially expressed metabolites (DEMs) (Figures S2). First, we screened the top 30 DEMs as shown in Figure 4A, and noteworthy findings unveiled the unmistakable presence of UA in cardiac tissues, including its secondary metabolites (Figures 4C and D). The KEGG pathway also revealed that the DEMs were mainly focused on lipid metabolism, especially in the top 30 DEMs, such as phosphatidyl choline, lysophosphatidylethanolamine, phosphatidyl glycerol, and phosphatidyl ethanolamine (Figures 4A, B, and F). Then, consistent with prior research,^31^ a reduction in long‐chain acylcarnitines, particularly l‐carnitine, were reduced and a boost in circulating free fatty acids (FFAs) and TGs were uncovered in the heart tissues of SACI mice (Figures 4E–G), underscoring a dysfunction in the conversion and utilization of FFAs in the SACI mouse hearts. These observations convey a disturbance in the capacity for fatty acid metabolism in SACI hearts. Intriguingly, UA administration proved to be effective in reversing the depletion of long‐chain acylcarnitines, while simultaneously lowering circulating levels of FFA and TG (Figures 4E–G).

To thoroughly explore the potential effects of UA on myocardial mitochondrial FAO, an in‐depth investigation of CPT1, a long‐chain acylcarnitine in FAO, was carried out, and the CPT1 inhibitor etomoxir (ETO) was employed. As depicted in Figures 4H and I, Western blotting analysis revealed that UA effectively reversed the suppression of CPT1 expression caused by SACI. However, the presence of ETO further intensified the inhibition of CPT1 and counteracted its upregulation by UA. Moreover, the expression levels of mitochondrial biogenesis and lipid metabolism‐related regulators (PPAR‐α, PGC1‐α, and CD36) were upregulated by UA (Figures 4H and I). All the findings strongly suggest that CPT1, a critical factor in FAO, holds significant potential as a target for UA.

### 2.5 UA attenuates cardiomyocyte injury by regulating CPT1

To ascertain the effectiveness of UA in diminishing cardiomyocyte injury in the context of SACI, primary mouse cardiomyocytes were extracted to facilitate our research investigation. Although the precise mechanisms of SACI remain unclear, it is evident that SAP multiorgan dysfunction and coinfection are intricately interconnected with sepsis.^32^ Therefore, to simulate SACI, we constructed a SICM model utilizing lipopolysaccharide (LPS). Next, we conducted a comprehensive assessment of the influence of UA on LPS under diverse concentrations and time intervals. Within the range of concentrations examined, the application of UA at 10 μg/mL demonstrated a notable enhancement in cell viability, effectively mitigating LPS‐induced SICM (Figure S3A). Subsequently, UA (10 μg/mL) interventions were performed at different time points. The application of UA intervention for 1, 2, and 4 h did not yield a substantial impact on cell survival compared with the LPS treatment. However, a notable elevation in cell survival was uncovered following the 6 h intervention. After 8 and 24 h of intervention, despite the observed increase in cell viability, no significant difference was detected (Figure S3B). In summary, we selected UA (10 μg/mL) intervention for 6 h as the UA treatment model.

**FIGURE 5:**
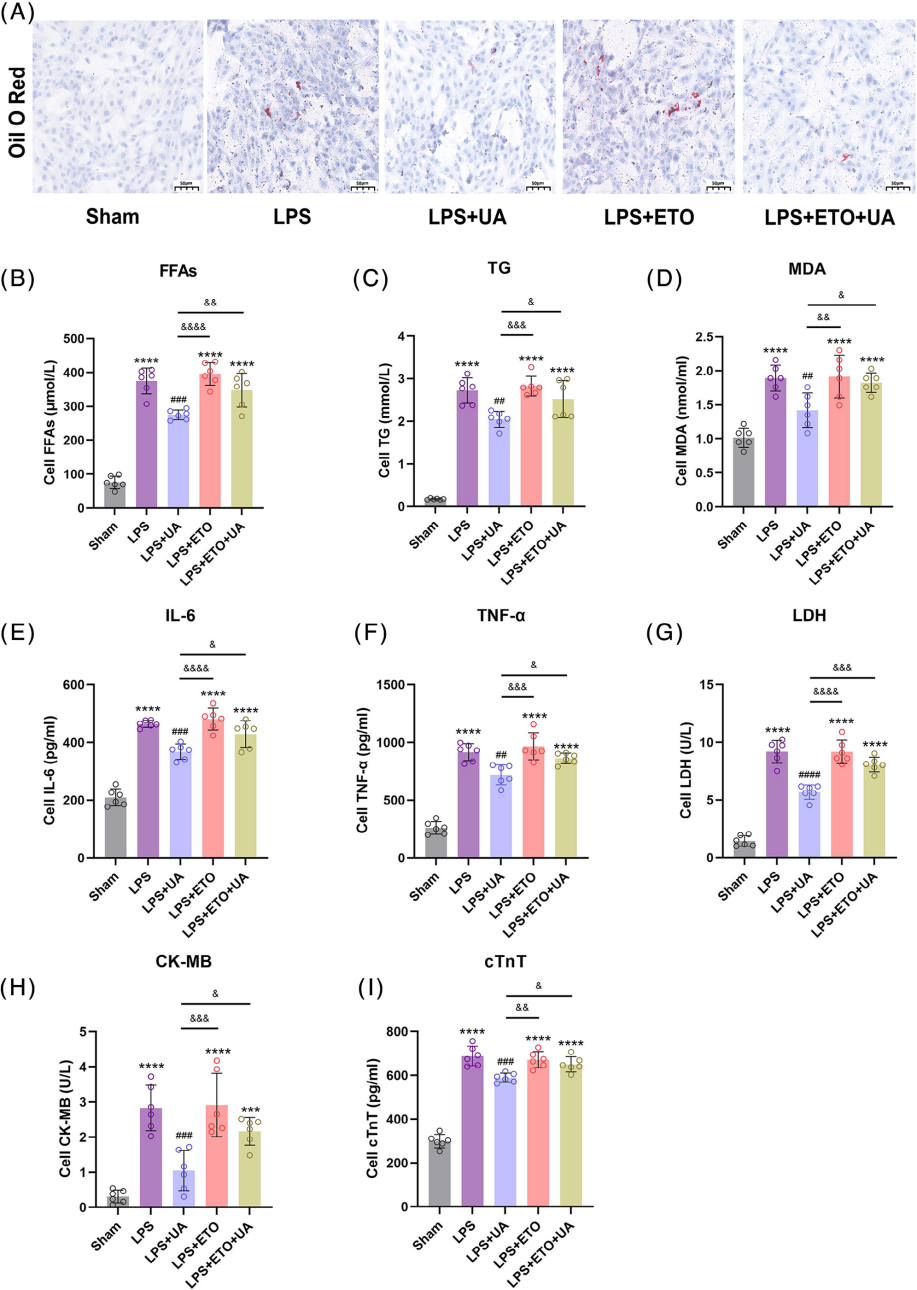
Urolithin A attenuates cardiomyocyte injury by regulating CPT1. (A) Representative images of oil red O (ORO) staining in primary cardiomyocytes in the sham group (sham), LPS‐induced injured group (LPS), and LPS plus Urolithin A treatment group (LPS+UA), LPS plus etomoxir inhibitor group (LPS+ETO), LPS plus Urolithin A treatment and etomoxir inhibitor group (LPS+ETO+UA) (scale bar = 50 μm). (B and C) Free fatty acid and triglyceride levels in the supernatant of primary cardiomyocytes in each group (*n* = 6). (D) MDA levels in the supernatant of primary cardiomyocytes in each group (*n* = 6). (E–I) IL‐6, TNF‐α, LDH, CK‐MB, and cTnT levels in the supernatant of primary cardiomyocytes in each group (*n* = 6). The results are presented as the mean ± SD. ***p < 0.005, ****p < 0.0001 vs. sham group; ##*p* < 0.01, ###*p* < 0.005, ####*p* < 0.0001 vs. LPS group, &*p* < 0.05, &&*p* < 0.01, &&&*p* < 0.005, &&&&*p* < 0.0001 vs. LPS+UA group, by one‐way ANOVA tests followed by Tukey tests.

Subsequently, the cells were partitioned into five distinct in vitro groups: (a) sham group: blank control group where cells were not treated in any way; (b) LPS group: LPS (1 μg/mL) intervention for 24 h; (c) LPS+UA group: LPS+UA (10 μg/mL, intervention for 6 h); (d) LPS+ETO group: LPS+ETO (100 μmol/L,^33^, ^34^ 6 h); (e) LPS+ETO+UA group: LPS+ETO (100 μmol/L, intervention for 6 h)+UA (10 μg/mL, intervention for 6 h). First, we found by Western blotting analysis that in primary cardiomyocytes CPT1 expression was reduced in the LPS group, and UA effectively upregulated its expression, but this ability was inhibited by ETO (Figure S4A). Next, as shown in Figure 5A, the employment of oil red O (ORO) staining demonstrated that UA mitigated the lipid accumulation induced by LPS, yet the efficacy of UA was abolished by ETO. Similarly, the inhibition of CPT1 by ETO exacerbated the LPS‐induced increases in the levels of FFAs and TGs in cardiomyocytes and promoted myocardial malondialdehyde (MDA) production, which indicates increased lipolysis, due to the inhibition of CPT1 by ETO, similarly diminished the ameliorative effect of UA (Figures 5B–D). In addition, inhibition of CPT1 similarly exacerbated LPS‐induced pathological myocardial injury, and upregulated the levels of the proinflammatory factors IL‐6 and TNF‐α and the cardiac injury markers LDH, CK‐MB, and cTnT in cardiomyocytes, considering its elimination of the therapeutic effect of UA on the heart of SACI (Figures 5E–I). Notably, in cardiomyocytes, we similarly found that UA inhibited the expression of the proapoptotic proteins Bax and Cleaved‐Caspase3, and upregulated the expression of the antiapoptotic protein Bcl‐2, but was affected by ETO, suggesting once again that inhibition of CPT1 attenuated the protective effect of UA on damaged cardiomyocytes (Figure S4B). In conclusion, these findings suggest that UA could potentially attenuate myocardial lipotoxicity by regulating CPT1.

### 2.6 UA improves mitochondrial energy metabolism in cardiomyocytes by modulating CPT1

**FIGURE 6:**
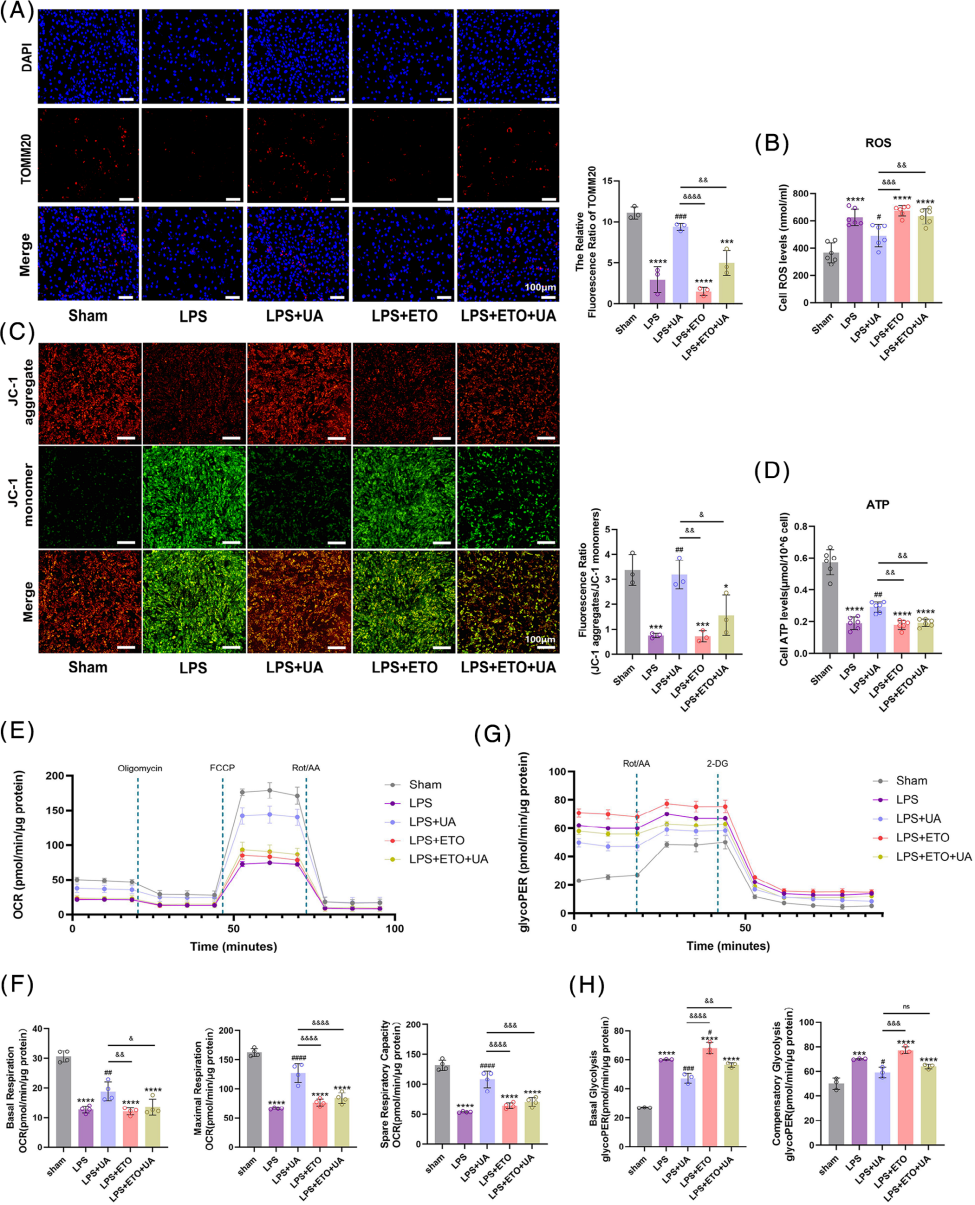
Urolithin A improves mitochondrial energy metabolism in cardiomyocytes by modulating CPT1. (A) Representative images of TOMM20 immunofluorescence staining of primary cardiomyocytes in the sham, LPS, LPS+UA, LPS+ETO, LPS+ETO+UA groups (scale bar = 100 μm; *n* = 3). (B) Changes in total ROS levels of primary cardiomyocytes in each group (*n* = 6). (C) Fluorescence images of JC‐1 mitochondrial membrane potential (Δ*Ψ*_m_) in cardiomyocytes (red: normal Δ*Ψ*_m_; green: decreased Δ*Ψ*_m_; blue: DAPI staining of cell nuclei; scale bar = 100 μm; *n* = 3). (D) Changes in total ATP levels of primary cardiomyocytes in each group (*n* = 6). (E) Schematic image of oxygen consumption rate (OCR) as determined by a Seahorse XFe24 Extracellular Flux Analyzer. (F) The levels of basal respiration, maximal respiration, and spare respiratory capacity in each group (*n* = 4). (G) Schematic image of glycolytic rate (GlycoPER) as determined by a Seahorse XFe24 Extracellular Flux Analyzer. (H) The levels of basal glycolysis and compensatory glycolytic in each group (*n* = 3). All measured data were normalized by total cellular protein. The results are presented as the mean ± SD. ***p < 0.005, ****p < 0.0001 vs. sham group; #*p* < 0.05, ##*p* < 0.01, ###*p* < 0.005, ####*p* < 0.0001 vs. LPS group, &*p* < 0.05, &&*p* < 0.01, &&&*p* < 0.005, &&&&*p* < 0.0001 vs. LPS+UA group, by one‐way ANOVA tests followed by Tukey tests.

For a more comprehensive understanding of the mechanistic contribution of UA to myocardial mitochondrial FAO, mitochondrial function and mitochondrial oxidative metabolism capacity were assessed. TOMM20, positioned within the outer mitochondrial membrane, is an indispensable marker for mitochondria, responsible for safeguarding mitochondrial structure and enabling proper functioning.^29^ Immunofluorescence analysis revealed that UA treatment resulted in an increase in the abundance of TOMM20, while the presence of ETO counteracted the influence of UA (Figure 6A). Similarly, inhibiting CPT1 increased the disruption of the Δ*Ψ*_m_, LPS‐induced ROS production, and ATP consumption in cardiomyocytes, which undermined the protective effect of UA on mitochondrial function (Figures 6B–D). An additional evaluation of mitochondrial oxidative metabolism capacity in cardiomyocytes was conducted using the Seahorse analyzer XFe24. The results indicated that inhibiting CPT1 markedly attenuated oxygen consumption rate (OCR) in primary cardiomyocytes (Figure 6E); this hindered the enhancing effect of UA on mitochondrial oxidative capacity, such as maximal respiration, basal respiration, and spare respiratory capacity (Figure 6F). These findings emphasize the indispensable role of CPT1 in the process by which UA restores the mitochondrial oxidative metabolism ability in cardiomyocytes.

Intriguingly, in contrast to that of the LPS group, the oxidative capacity of the ETO group was not lower, which may be attributed to cellular compensation under conditions of stress. However, UA was still able to restore the impaired mitochondrial oxidative capacity induced by ETO (Figure 6F). In this regard, the glycolytic capacity of cardiomyocytes was further explored. Interestingly, the inhibition of FAO by ETO resulted in an abnormally elevated glycolytic rate (Figure 6G), and cells damaged by LPS simultaneously exhibited augmented levels of basal glycolysis and compensatory glycolytic capacity (Figure 6H). Nevertheless, UA exerted a restorative effect on the abnormal glycolytic rate of cardiomyocytes to some extent (Figures 6G and H).

## 3 DISCUSSION

The findings of this study elucidate the robust protective properties of UA, a natural compound, against SAP‐induced cardiac injury. Analysis of SACI mouse heart transcriptome profiling revealed the restoration of mitochondrial function as a central pathway through which UA effectively combats SACI. More in‐depth investigations showed that UA intervention could impact the FAO‐related gene CPT1 in mouse hearts to reprogram cardiac metabolism, promote energy metabolism, diminish oxidative stress, restore mitochondrial dysfunction, and alleviate myocardial apoptosis.

The pathogenesis of SACI is extraordinarily complex and inconclusive, as underscored by current studies, SAP‐induced multiorgan failure has intimate correlation with SIRS, and the signaling cascade response caused by inflammatory factors such as IL‐6, TNF‐α is one of the main factors.^7^ In our investigation, we observed heightened inflammation and histopathological injury in the cardiac tissue, alongside raised inflammatory markers’ levels including TNF‐α and IL‐6, in a mouse model of SACI. In keeping with past investigations,^35^, ^36^ our study also unmasked elevated concentrations of LDH, CK‐MB, BNP, cTnT, and cTnI, in a mouse model of SACI, conveying that severe cardiac injury occurred in SACI mice.

Findings from recent studies reveal the anti‐inflammatory potential of the natural compound UA. It downregulates inflammatory factors, such as IL‐6 and TNF‐α, and downregulates cardiac injury markers, such as CK‐MB and LDH levels, to exert cardioprotective effects.^24^, ^37^ Likewise, our findings demonstrated a significant inhibition of the upregulation of inflammatory factors, including TNF‐α and IL‐6 levels, by UA. Furthermore, UA effectively decreased the serum levels of serum LDH, CK‐MB, BNP, cTnT, and cTnI, and the pathological features of myocardial tissues in SACI mice were prominently lower after UA treatment. Notably, UA significantly reduced pancreatic lipase and amylase levels and repaired damaged pancreatic tissues, suggesting that the inflammatory ameliorating effect of UA on the pancreas itself may be one of the mechanisms of action of its protection against SACI. Similar to previous findings,^28^, ^38^ our transcriptome analysis provided compelling evidence that UA elicited a similar upregulation of the mitochondrial gene set in the hearts of SACI mice, with a particular emphasis on mitochondrial biogenesis and oxidative phosphorylation. Notably, UA restored Δ*Ψ*_m_ and ATP production in wounded hearts while effectively alleviating mitochondrial swelling and structural disruptions in SACI hearts. Furthermore, based on the mitochondrial apoptotic pathway, malfunctioning mitochondria can instigate the intrinsic mechanism of cardiomyocyte apoptosis.^30^, ^39^ Prior studies have discovered that UA has the ability to mitigate left ventricular apoptosis,^24^ and our results showed that UA effectively diminished myocardial apoptosis in SACI mice through the downregulation of proapoptotic factors, such as Bax and Cleaved‐Caspase3 and the upregulation of the antiapoptotic factor Bcl‐2. The presented evidence substantiates the cardioprotective effects of UA in SACI mice, highlighting its role in restoring mitochondrial function.

As previously mentioned, the utilization of fatty acids for oxidative metabolism represents a primary mechanism by which cardiac mitochondria produce ATP.^40^ FFAs are taken up into the cytoplasm by fatty acid translocase/cluster of differentiation (FAT/CD36) to form acyl coenzyme A (CoA), which functions by entering the mitochondria via CPT1. It eventually participates in fatty acid β‐oxidation and may also participate in phospholipid synthesis.^40^ Therefore, we unveiled a decrease in long‐chain acylcarnitine levels and an increase in phospholipid synthesis in SACI mouse hearts. Serum FFA and TG levels were elevated, possibly as a result of disturbed lipolysis and fatty acid beta following an episode of SACI,^41^ which was restored by UA. Given that CPT1 serves as a crucial enzyme for FAO in cardiac injury,^14^ we hypothesized that UA restores the FAO of SACI by regulating CPT1. As part of our study design, a high dose of ETO was included as a comparative control. As previously described,^42^ ETO, acting as a CPT1 inhibitor, efficiently inhibits FAO, and elicits unintended effects on mitochondria, leading to the perturbation of mitochondrial function. In our study, we revealed that ETO significantly inhibited CPT1 expression in SACI, and this was not reversed by UA. These findings suggest that CPT1 may be a therapeutic target for UA. Additionally, the injured heart exhibited inhibited expression of key factors involved in FAO, such as PPAR‐α and PGC1‐α, which is a critical transcriptional regulator of mitochondrial biogenesis, and the fatty acid transporter protein CD36.^15^, ^16^, ^17^ Our study additionally revealed that their expression was inhibited by SACI, while UA efficiently restored the expression of these metabolic genes. Despite the similar reduction observed with ETO, UA exhibited a distinct ability to counteract this reduction and restore the expression of these factors. Consequently, UA may target CPT1 and upregulate related genes to exert a promising therapeutic effect on SACI.

Given the convergence in the mechanistic pathways between SICM and SACI, we induced primary cardiomyocytes with LPS in vitro experiments as an attempt to simulate the myocardial damage in the SACI condition.^43^, ^44^ Inhibition of FAO instigates metabolic reprogramming, disturbing the equilibrium in substrate utilization and leading to the accumulation of excess TGs in the myocardium.^10^, ^45^ This also inhibits mitochondrial function,^12^ fosters apoptosis,^46^ induces cellular stress, and contributes to the occurrence of arrhythmias,^47^ ultimately exacerbating myocardial injury^48^ as a phenomenon known as cardiac lipotoxicity. Supporting our data, UA reduces TG accumulation and restores this mitochondrial dysfunction.^26^, ^49^ Our fruit findings also revealed that high doses of ETO suppressed fatty acid metabolism, exerting a substantial elevation in MDA levels in cardiomyocytes. This inhibition further intensified myocardial lipid accumulation and inflammatory damage, exacerbated mitochondrial dysfunction, triggered excessive generation of ROS, depleted ATP, disrupted Δ*Ψ*_m_, and to a certain degree, weakened the protective effects of UA on cardiomyocytes and mitochondria. Furthermore, we found that UA possessed the capacity to restore mitochondrial respiratory capacity in cardiomyocytes impaired after LPS induction and restore a portion of mitochondrial oxidative phosphorylation. Notably, while it has been suggested that SICM is associated with a decline in FAO, an intriguing finding is the absence of compensatory upregulation in glucose oxidation, likely due to the presence of insulin resistance.^46^, ^50^, ^51^ However, in the context of the Randall cycle, the diminished utilization of fatty acids can possibly give rise to reactivation of glucose oxidation.^52^, ^53^ Likewise, evidence derived from an experimental sepsis model proposed that enhanced glycolytic metabolism might be a contributing factor to cardiac insufficiency in sepsis.^54^ In a similar vein, our analysis validated that the rate of glycolysis was increased in LPS‐induced cardiomyocytes, and its observed abnormal increase in glycolysis in LPS‐induced cardiomyocytes can be viewed as a compensatory response, partially mitigating the inhibited FAO induced by the addition of the CPT1 inhibitor ETO. More importantly, we demonstrated that this energy metabolism disorder could be restored by the modulation of CPT1 by UA.

## 4 CONCLUSIONS

In summary, our investigation reveals the significant protective properties of the natural compound UA against SACI. The underlying mechanism for this action could be attributed to the ability of UA to attenuate cardiac mitochondrial dysfunction and reduce myocardial apoptosis by restoring the homeostasis of mitochondrial fatty acid oxidative metabolism. CPT1 presents itself as a potential target for balancing mitochondrial fatty acid metabolism in SACI.

## 5 MATERIALS AND METHODS

### 5.1 Antibodies and reagents

Taurocholic acid sodium salt hydrate (T4009) and LPS (L2880) were purchased from Sigma–Aldrich. UA (S5312) and ETO sodium salt (S8244) were purchased from Selleck Chemicals. Anti‐CD36 (21277‐1‐AP) and anti‐PGC1‐α (66369‐1‐lg) were procured from Proteintech Company. Anti‐Bcl‐2 (bs‐4563R) was purchased from Bioss. Anti‐Bax (AF0120), anti‐COX I (AF7002), and anti‐NDUFC1 (DF9667) were procured from Affinity Biosciences. Anti‐CPT1 (A20746) and anti‐TOMM7 (A17711) were purchased from ABclonal Technology. Anti‐PPAR‐α (sc‐398394) was purchased from Santa Cruz Biotechnology. Anti‐COX IV (ab202554), anti‐Cleaved‐Caspase3 (ab214430) and β‐actin antibody (ab8226) were purchased from Abcam. GAPDH antibody (KC‐5G5) were purchased from Aksomics. The anti‐TNF‐α (60291‐1‐lg) and anti‐Bcl‐2 (26593‐1‐AP) used for the immunohistochemistry were procured from Proteintech. Anti‐Cleaved‐Caspase3 (9661S) used for the immunohistochemistry was purchased from Cell Signaling. Anti‐IL‐6 (A1570) used for the immunohistochemistry was purchased from ABclonal Technology. Anti‐TOMM20 (11802−1‐AP) used for the immunofluorescence was purchased from Proteintech. The mouse IL‐6 (88‐7064) and TNF‐α (88‐7324) enzyme‐linked immunosorbent assay (ELISA) kits were obtained from ThermoFisher Scientific. The mouse cTnT assay kit (H149‐4) was purchased from Jiancheng. FFAs assay kit (BC0590) was obtained from Solarbio. MDA assay kit (S0131S) was obtained from Beyotime. cTnI assay kit (RX202543M) and BNP assay kit (RX202726M) were obtained from RuxinBio.

### 5.2 Animals

The Chengdu Dashuo Biological Technology Co., Ltd. supplied the male C57BL/6 mice (20 ± 2 g) for the study. The housing conditions maintained for the mice adhered to a specific‐pathogen‐free (SPF) environment (humidity 50 ± 5%; temperature 20 ± 2°C). Approval for the experiments was granted by the Experimental Animal Ethics Committee of the Sichuan University of West China Hospital (Approval Number: 20230112001), and all protocols undertaken in this study meticulously followed the principles elucidated in the Guide for the Care and Use of Laboratory Animals, as stipulated by the National Institutes of Health (NIH Publication no. 86‐23, revised in 1996).

### 5.3 Mouse SACI model establishment and treatment

The mice underwent adaptive feeding under standard conditions for one week and were subsequently fasted for 12 h prior to the experiment. The mice were assigned to five groups following the random number table method: sham group, SACI group, SACI plus UA treatment group (UA group), SACI plus ETO inhibitor group (SACI+ETO group), and SACI plus UA treatment and ETO inhibitor group (SACI+ETO+UA group). There were nine mice per group. Intraperitoneal injection of 2% sodium pentobarbital (50 mg/kg body weight) was administered to anaesthetize all mice. As previously mentioned,^55^ upon opening the abdomen, we located the opening of the pancreaticobiliary duct in the descending duodenum and proceeded to apply arterial clamping to secure the hepatic duct. To induce SACI, a 2% taurocholic acid sodium salt solution (0.1 mL/100 g body weight) was retrogradely perfused via the pancreaticobiliary duct, adopting a perfusion pump (LD‐P2020II; Lande Medical Appliance Co., Ltd.) ensuring a consistent velocity (0.06 mL/h, 4 min).^56^ An equivalent amount of normal saline was injected into the sham group as a control measure. UA (30 mg/kg, dissolved in 0.1% dimethyl sulfoxide) was administered via intraperitoneal injection to the UA experimental cohort, as per the previous methodology.^57^ Five distinct intraperitoneal injection time points were chosen to establish the optimal treatment time: 3 or 1 h prior to SACI induction, and 1, 3, or 6 h after SACI induction. ETO (15 mg/kg i.p.)^58^ was given 1 h after SACI induction. Moreover, the sham and SACI groups received equivalent quantities of saline concurrently with the ETO or UA injection. Following a 24‐h period, arterial blood, heart, and pancreatic tissues were promptly collected. Fresh heart tissues were utilized for experiments related to mitochondria, while the remaining samples were stored at −80°C for subsequent analysis.

### 5.4 Mouse primary cardiomyocyte culture

According to the previous approach,^59^ the decontamination of specifically chosen neonatal C57BL/6 mice, aged between 1 and 3 days, was accomplished by immersing them in a solution of 75% ethanol. The hearts were then extracted by opening the chest and placed in pre‐cooled PBS. Removal of the connective tissue at the cardiac base was performed, followed by dissection and thorough washing of the apical tissue with PBS, ensuring a minimum of three wash cycles. Following this, digestion was initiated by adding a minimum of three times the volume of digestion solution (containing 0.04% type II collagenase (17101015; Gibco) and 0.08% trypsin (1734858; Gibco)) in a water bath with a fixed temperature set at 37°C for a duration of 10 min. Following the removal of the supernatant, the digestion solution was introduced and allowed to incubate for 8 min. The digestion process was terminated by adding complete medium (myocardial medium (SC01831008; FenghuiShengwu) with 10% FBS (1414426; Gibco) and 1% penicillin/streptomycin (15070063; Gibco)). The described procedure was repeated in successive cycles until the tissue digestion reached completion. Afterward, the supernatant solution was filtered through 100 μm, followed by 300×*g* centrifugation for 5 min. The subsequent supernatant was discarded, and the remaining solution was evenly spread on a petri dish. This dish was then transferred to a 37°C, 5% CO_2_ incubator for two cycles of differential wall attachment, with each cycle lasting 50−60 min.

### 5.5 Screening of multigradient UA concentrations and CCK‐8 cell proliferation assay

When the cell culture attained 80% confluence, the existing medium was exchanged with a fresh complete medium fortified with 1 μg/mL of LPS (Sigma–Aldrich). The cells were then incubated for a duration of 24 h.^60^ After establishing the LPS model induction, interventions were carried out with multiple gradient concentrations of UA at 1,2,5,10,20 μg/mL and assayed at different time points to identify the optimal the best UA intervention model.^61^

The Cell Counting Kit‐8 kit (CA1210‐1000; Solarbio) was utilized to detect the viability of primary cardiomyocytes. Cells grouped above were inoculated in 96‐well plates with different conditions of intervention, and 10 L of CCK‐8 solution was injected into each well. Cells were then cultured in the solution for 2 h at a temperature of 37°C and 5% CO_2_. In the end, a spectrophotometer was employed to measure the absorbance at 450 nm.

### 5.6 Detection of physiological and biochemical indexes

Following the collection of arterial blood from mouse hearts, the supernatant was obtained by subjecting it to centrifugation (5000×*g*, 10 min, 4°C). Pancreatic and heart tissues harvested from mice or cell supernatants collected from six‐well plates. Subsequently, these samples were stored in a freezer at −80°C. Utilizing an automated biochemical analyzer (Chemray240; Rayto), the content of lipase and amylase in the serum, along with indicators of myocardial injury and blood lipid indexes such as LDH, TGs, and the isoenzyme of CK‐MB, were identified. TNF‐α, IL‐6, cTnT, FFA, and MDA levels were assessed by ELISA.^62^

### 5.7 Histopathological examination

Following 24 h of fixation at room temperature using 4% paraformaldehyde, the pancreatic and heart tissues underwent paraffin embedding and were then sliced into sections measuring 4 μm in thickness. The tissues were subjected to haematoxylin and eosin staining at the surrounding temperature employing the H&E Staining kit (cat. no. C0105; Beyotime Institute of Biotechnology). Hematoxylin staining was performed for 5 min, followed by eosin staining for 1 min. The stained sections were imaged using Panoramic Digital Slide Scanners manufactured by 3D HISTECH. Ten fields were randomly selected to observe histopathological changes under the microscope. As previously described,^63^, ^64^ the degree of pancreatic and cardiac tissues damage was assessed.

### 5.8 Immunohistochemistry

After dewaxing and dehydration using xylene and ethanol, the myocardial tissues were repaired with sodium citrate. Following this, incubation with 3% hydrogen peroxide and bovine serum albumin (BSA) was performed to block endogenous peroxidase activity and antigen. Afterward, the cardiac tissues were coincubated with the primary antibodies overnight at 4°C. Primary antibodies, including those against TNF‐α (dilution 1:500; Proteintech), Bcl‐2 (dilution 1:500; Proteintech), IL‐6 (dilution 1:500; ABclonal), and Cleaved‐Caspase3 (dilution 1:100, CST), were employed. After rinsing with PBS, the appropriate secondary antibody was added, and the cells were cultured at 37°C for 1 h. Subsequently, the color development process utilized DAB color development solution (ab64238; Abcam), and hematoxylin was applied for counterstaining. The 3DHISTECH panoramic SCAN instrument was implemented for imaging and observation. Analysis of the findings was carried out using the National Institutes of Health's ImageJ software.

### 5.9 Immunofluorescence

To process the cells or frozen sections of heart tissues, a fixation step was executed using 4% paraformaldehyde for 15 min. Subsequently, permeabilization was achieved with 0.5% Triton X‐100 for a duration of 2 min, followed by blocking with 5% BSA. Incubation of the sections with the primary antibody TOMM20 (1:100 dilution; Proteintech) was carried out overnight at 4°C. The addition of secondary antibodies was followed by the use of DAPI (G1012‐10ML; Servicebio) as a counterstain for the nucleus. The images were captured with the assistance of a fluorescence microscope (Olympus).

### 5.10 Transmission electron microscopy

Heart tissues were subjected to prefixation using a 3% glutaraldehyde solution to enable TEM observation. Subsequently, postfixation was performed by applying 1% osmium tetroxide. Tissues were then dehydrated through a series of acetone solutions, infiltrated with Epox 812, and eventually embedded. To visualize the semithin sections, methylene blue staining was employed. Using a diamond knife, ultrathin sections were sliced and subsequently treated with uranyl acetate and lead citrate staining. The acquired sections were inspected utilizing a JEM‐1400‐FLASH transmission electron microscope.

### 5.11 ORO staining

In keeping with the manufacturer's instructions, ORO (#O0625; Sigma) staining was conducted on frozen heart sections obtained from diverse groups of mice.

### 5.12 Terminal deoxynucleotidyl transferase‐mediated dUTP nick‐end labeling immunofluorescence

Transferase‐mediated dUTP nick‐end labeling (TUNEL) staining of frozen mouse heart sections was performed using a cell death detection kit (Millipore) according to the instructions provided by the manufacturer. DAPI (G1012‐10ML; Servicebio) was used to stain the nuclei.

### 5.13 Mitochondrial function assays

#### 5.13.1 | Mitochondrial membrane potential

To gauge the degree of mitochondrial dysfunction, mitochondria were isolated from fresh heart tissues using the Mitochondrial Extraction Kit (SM0020; Solarbio) according to the manufacturer's instructions. A Mitochondrial Membrane Potential Assay (JC‐1) Kit (M8650; Solarbio) was employed to assay the extracted fresh heart mitochondria, following the provided instructions, and the acquired data were measured with a fluorescence microplate reader (SynergyMx; BioTeK). Six‐well plates yielded an ample supply of primary cardiomyocytes, with each group being subjected to JC‐1 dye staining for 20 min at 37°C. Next, images were captured using a fully automated inverted fluorescence microscope (Olympus IX83; Olympus Corporation). Aggregated JC‐1 (red fluorescence) indicated normal cardiomyocytes, while monomeric JC‐1 (green fluorescence) indicated cells in which Δ*Ψ*_m_ was decreased.

#### 5.13.2 | ATP and intracellular ROS levels

According to the instructions, the ATP content and ROS levels in fresh heart tissues or primary cardiomyocytes were confirmed utilizing the ATP Content Assay Kit (BC0300; Solarbio) and the Reactive Oxygen Species Assay Kit (S0033S; Beyotime). A fluorescence microplate reader (SynergyMx; BioTeK) was utilized for the measurement process.

### 5.14 Western blotting

After centrifugation at 12,000×*g* for 5 min, homogenization of tissues or cells in RIPA buffer was carried out to extract protein. To ensure the standardization of proteins in each lane, the extracted protein samples were quantified using a BCA kit (P0012S; Beyotime). Supernatant protein (20–40 μg/lane) was separated by 12% SDS‐PAGE. Upon electrophoresis, the proteins were transferred onto polyvinylidene difluoride membranes (Millipore). The membranes were submerged in commercial blocking solution (EpiZyme Biotech) and shaken on a shaker at room temperature for 10 min. The membranes were washed three times with TBST and subsequently incubated overnight at 4°C with primary antibodies, including COX I (1:2000; Affinity), COX IV (1:2000; Abcam), NDUFC1 (1:3000; Affinity), TOMM7 (1:5000; ABclonal), Bax (1:1000; Affinity), Bcl‐2 (1:1000; Bioss), Cleaved‐Caspase3 (1:1000; Abcam), CD36 (1:1000; Proteintech), PGC1‐α (1:50,000; Proteintech), CPT1 (1:1000; ABclonal), PPAR‐α (1:1000; Santa Cruz), GAPDH(1:10,000; Aksomics), and β‐actin (1:10,000, Abcam). After being rinsed thrice with TBST, the membranes underwent a 60‐min incubation period with the corresponding secondary antibody, which was labelled with horseradish peroxidase. Following three additional washes, the blots were rendered visible employing a cutting‐edge imaging system from Bio‐Rad and subsequently subjected to analysis through the utilization of ImageJ software (National Institutes of Health).

### 5.15 RNA‐sequencing

Adhering to the instructions provided by the manufacturer, the employment of TRIzol reagent (Invitrogen) facilitated the extraction of total RNA from heart tissues. The extracted total RNA was assessed for RNA integrity using the Agilent Bioanalyzer 2100 (Agilent Technologies). Utilizing a Qubit® 3.0 Fluorometer (Life Technologies) and Nanodrop One spectrophotometer (Thermo Fisher Scientific Inc.), the concentration and purity of the total RNA were measured. Majorbio Biotech performed the library preparation and sequencing steps on an Illumina HiSeq Xten/NovaSeq 6000, meticulously following the standard procedures in the cBot User Guide manual. The data advanced analysis was performed online in APExBIO (https://analize.cloud.apexbio.cn/).

### 5.16 Untargeted metabolomics

Each sample, weighing approximately 50 mg and enclosed within an EP tube, was subjected to further analysis. Sample preparation was carried out according to the instructions. LC/MS detection was achieved by employing the Agilent 1290 Infinity UPLC system, which was accompanied by the Agilent 6545 UHD and Accurate‐Mass Q‐TOF mass spectrometer. Adhering to the prescribed instructions from the manufacturer, a meticulous injection of 4 μL of samples was carried out onto a Waters XSelect HSS T3 column (2.5 μm, 100 × 2.1 mm). Mass spectrometry was conducted by employing positive ion mode in conjunction with negative ion mode. A range of *m*/*z* 50−1000 was selected for the acquisition mass range during the analysis. Utilizing Agilent Masshunter Qualitative Analysis B.08.00 software (Agilent Technologies), the initial data were transformed into a common (mz.data) format. For subsequent processing tasks, such as peak identification, retention time calibration, autointegration, and normalization, the XCMS package within the R software platform was employed. During normalization, the original peak area was categorized by the corresponding internal standard peak area. For the end, the utilization of the Human Metabolome Database (HMDB) at http://www.hmdb.ca facilitated the process of metabolite matching. The data advanced analysis was performed online in APExBIO (https://analize.cloud.apexbio.cn/).

### 5.17 Seahorse metabolic flux analysis

As per the manufacturer's protocol, the Seahorse XFe24 Extracellular Flux Analyser was employed for a stepwise addition of specific compounds obtained from the Seahorse XF Cell Mitochondrial Stress Test Kit and XF Glycolytic Rate Assay Kit (#103015‐100, 103344‐100; Seahorse Bioscience) to enable the measurement and calculation of the OCR and extracellular acidification rate. In a concise manner, Seahorse XFe24 cell culture plates received primary cardiomyocytes at a density of 2 × 10^4^ cells per well, followed by a 24 h incubation duration. Subsequently, cells underwent a 24‐h pretreatment with LPS (1 μg/mL), followed by a 6‐h cotreatment with either UA (10 μg/mL) or ETO (10 μg/mL) in the drug experimental groups. In line with the preceding information,^65^ prior to the assay, a preassay step consisted of the incubation of cells with Seahorse XF basal medium (containing 1 mM pyruvate, 2 mM glutamine, and 10 mM glucose, pH 7.4) at 37°C for 1 h in a CO_2_‐free incubator. Sequential addition of oligomycin (1.0 μM), FCCP (1.0 μM), and rotenone/antimycin A (0.5 μM) was performed during the cell OCR assay to determine the maximum respiration, ATP‐linked OCR, basal mitochondrial respiration, and spare respiratory capacity of the cells. To determine the basal glycolysis and glycolytic proton efflux rate (glycoPER) of the cells, oligomycin (0.5 μM) and 2‐DG (50 mM) were added sequentially in the glycolytic rate assay. Subsequently, all recorded values were normalized to proteins (pmol O_2_/min/μg protein) relying on BCA protein assay analysis.

### 5.18 Statistical analysis

Data are expressed as the mean ± SD and tested for normality. To compare variations among multiple groups in both in vivo and in vitro investigations, a one‐way analysis of variance (ANOVA) was conducted, followed by Tukey's test for diverse comparisons using GraphPad Prism version 9 software (GraphPad Prism Software). *p* < 0.05 was deemed to indicate a statistically significant difference.

## AUTHOR CONTRIBUTIONS

Meihua Wan and Wenfu Tang designed the experiments. Yue Yang and Qian Hu performed the experiments. Yue Yang, Hongxin Kang, and Juan Li drafted the manuscript, analyzed, and interpreted the data. Xianlin Zhao and Lv Zhu assisted in data processing. Meihua Wan and Wenfu Tang revised the manuscript. All authors read and approved the final version of the manuscript. All authors agree to take responsibility for all aspects of the work to ensure integrity and accuracy. All authors have read and approved the final manuscript.

## CONFLICT OF INTEREST STATEMENT

The authors have no conflict of interest to declare.

## ETHICS STATEMENT

Approval for the experiments was granted by the Experimental Animal Ethics Committee of the Sichuan University of West China Hospital (Approval Number: 20230112001), and all protocols undertaken in this study meticulously complied with the national and international guidelines for the Care and Use of Laboratory Animals.

## Supporting information

Supporting Information

## Data Availability

All data generated or analyzed in this study are available from the corresponding author on reasonable request.

## References

[1] Banks PA , Bollen TL , Dervenis C , et al. Classification of acute pancreatitis–2012: revision of the Atlanta classification and definitions by international consensus. Gut. 2013;62:102‐111. 23100216 10.1136/gutjnl-2012-302779

[2] Leppäniemi A , Tolonen M , Tarasconi A , et al. 2019 WSES guidelines for the management of severe acute pancreatitis. World J Emerg Surg. 2019;14:27. 31210778 10.1186/s13017-019-0247-0 PMC6567462

[3] Yegneswaran B , Kostis JB , Pitchumoni CS . Cardiovascular manifestations of acute pancreatitis. J Crit Care. 2011;26:225. e211‐228. 10.1016/j.jcrc.2010.10.013 21185146

[4] Calleja GA , Barkin JS . Acute pancreatitis. Med Clin North Am. 1993;77:1037‐1056. 7690443 10.1016/s0025-7125(16)30209-7

[5] Vasantha Kumar A , Mohan Reddy G , Anirudh Kumar A . Acute pancreatitis complicated by acute myocardial infarction—a rare association. Indian Heart J. 2013;65:474‐477. 23993014 10.1016/j.ihj.2013.06.009 PMC3861136

[6] Nadkarni N , Bhasin DK , Rana SS , et al. Diastolic dysfunction, prolonged QTc interval and pericardial effusion as predictors of mortality in acute pancreatitis. J Gastroenterol Hepatol. 2012;27:1576‐1580. 22849657 10.1111/j.1440-1746.2012.07229.x

[7] Luo Y , Li Z , Ge P , et al. Comprehensive mechanism, novel markers and multidisciplinary treatment of severe acute pancreatitis‐associated cardiac injury—a narrative review. J Inflamm Res. 2021;14:3145‐3169. 34285540 10.2147/JIR.S310990 PMC8286248

[8] Kellner A , Robertson T . Selective necrosis of cardiac and skeletal muscle induced experimentally by means of proteolytic enzyme solutions given intravenously. J Exp Med. 1954;99:387‐404. 13152283 10.1084/jem.99.4.387 PMC2136237

[9] Ammori BJ , Leeder PC , King RF , et al. Early increase in intestinal permeability in patients with severe acute pancreatitis: correlation with endotoxemia, organ failure, and mortality. J Gastrointest Surg. 1999;3:252‐262. 10481118 10.1016/s1091-255x(99)80067-5

[10] Bertero E , Maack C . Metabolic remodelling in heart failure. Nat Rev Cardiol. 2018;15:457‐470. 29915254 10.1038/s41569-018-0044-6

[11] D'Souza K , Nzirorera C , Kienesberger PC . Lipid metabolism and signaling in cardiac lipotoxicity. Biochim Biophys Acta. 2016;1861:1513‐1524. 26924249 10.1016/j.bbalip.2016.02.016

[12] Bugger H , Abel ED . Molecular mechanisms for myocardial mitochondrial dysfunction in the metabolic syndrome. Clin Sci (Lond). 2008;114:195‐210. 18184113 10.1042/CS20070166

[13] Unger RH , Orci L . Lipoapoptosis: its mechanism and its diseases. Biochim Biophys Acta. 2002;1585:202‐212. 12531555 10.1016/s1388-1981(02)00342-6

[14] Agrawal V , Hemnes AR , Shelburne NJ , et al. l‐Carnitine therapy improves right heart dysfunction through Cpt1‐dependent fatty acid oxidation. Pulm Circ. 2022;12:e12107. 35911183 10.1002/pul2.12107 PMC9326551

[15] Irie H , Krukenkamp IB , Brinkmann JF , et al. Myocardial recovery from ischemia is impaired in CD36‐null mice and restored by myocyte CD36 expression or medium‐chain fatty acids. Proc Natl Acad Sci USA. 2003;100:6819‐6824. 12746501 10.1073/pnas.1132094100 PMC164530

[16] Lopaschuk GD , Ussher JR , Folmes CD , et al. Myocardial fatty acid metabolism in health and disease. Physiol Rev. 2010;90:207‐258. 20086077 10.1152/physrev.00015.2009

[17] Standage SW , Waworuntu RL , Delaney MA , et al. Nonhematopoietic peroxisome proliferator‐activated receptor‐α protects against cardiac injury and enhances survival in experimental polymicrobial sepsis. Crit Care Med. 2016;44:e594‐603. 26757163 10.1097/CCM.0000000000001585 PMC4940302

[18] Cásedas G , Les F , Choya‐Foces C , et al. The metabolite urolithin‐a ameliorates oxidative stress in neuro‐2a cells, becoming a potential neuroprotective agent. Antioxidants (Basel). 2020;9:177. 32098107 10.3390/antiox9020177 PMC7070385

[19] Toney AM , Fox D , Chaidez V , et al. Immunomodulatory role of urolithin A on metabolic diseases. Biomedicines. 2021;9:192. 33671880 10.3390/biomedicines9020192 PMC7918969

[20] Cerdá B , Espín JC , Parra S , et al. The potent in vitro antioxidant ellagitannins from pomegranate juice are metabolised into bioavailable but poor antioxidant hydroxy‐6H‐dibenzopyran‐6‐one derivatives by the colonic microflora of healthy humans. Eur J Nutr. 2004;43:205‐220. 15309440 10.1007/s00394-004-0461-7

[21] Cerdá B , Tomás‐Barberán FA , Espín JC . Metabolism of antioxidant and chemopreventive ellagitannins from strawberries, raspberries, walnuts, and oak‐aged wine in humans: identification of biomarkers and individual variability. J Agric Food Chem. 2005;53:227‐235. 15656654 10.1021/jf049144d

[22] D'Amico D , Andreux PA , Valdés P , et al. Impact of the natural compound urolithin A on health, disease, and aging. Trends Mol Med. 2021;27:687‐699. 34030963 10.1016/j.molmed.2021.04.009

[23] Zhang Y , Aisker G , Dong H , et al. Urolithin A suppresses glucolipotoxicity‐induced ER stress and TXNIP/NLRP3/IL‐1β inflammation signal in pancreatic β cells by regulating AMPK and autophagy. Phytomedicine. 2021;93:153741. 34656886 10.1016/j.phymed.2021.153741

[24] Tang L , Mo Y , Li Y , et al. Urolithin A alleviates myocardial ischemia/reperfusion injury via PI3K/Akt pathway. Biochem Biophys Res Commun. 2017;486:774‐780. 28343995 10.1016/j.bbrc.2017.03.119

[25] Cui GH , Chen WQ , Shen ZY . Urolithin A shows anti‐atherosclerotic activity via activation of class B scavenger receptor and activation of Nef2 signaling pathway. Pharmacol Rep. 2018;70:519‐524. 29660655 10.1016/j.pharep.2017.04.020

[26] Savi M , Bocchi L , Mena P , et al. In vivo administration of urolithin A and B prevents the occurrence of cardiac dysfunction in streptozotocin‐induced diabetic rats. Cardiovasc Diabetol. 2017;16:80. 28683791 10.1186/s12933-017-0561-3 PMC5501434

[27] Kang I , Kim Y , Tomás‐Barberán FA , et al. Urolithin A, C, and D, but not iso‐urolithin A and urolithin B, attenuate triglyceride accumulation in human cultures of adipocytes and hepatocytes. Mol Nutr Food Res. 2016;60:1129‐1138. 26872561 10.1002/mnfr.201500796

[28] Andreux PA , Blanco‐Bose W , Ryu D , et al. The mitophagy activator urolithin A is safe and induces a molecular signature of improved mitochondrial and cellular health in humans. Nat Metab. 2019;1:595‐603. 32694802 10.1038/s42255-019-0073-4

[29] Harbauer AB , Zahedi RP , Sickmann A , et al. The protein import machinery of mitochondria‐a regulatory hub in metabolism, stress, and disease. Cell Metab. 2014;19:357‐372. 24561263 10.1016/j.cmet.2014.01.010

[30] Del Re DP , Amgalan D , Linkermann A , et al. Fundamental mechanisms of regulated cell death and implications for heart disease. Physiol Rev. 2019;99:1765‐1817. 31364924 10.1152/physrev.00022.2018 PMC6890986

[31] Ranjbarvaziri S , Kooiker KB , Ellenberger M , et al. Altered cardiac energetics and mitochondrial dysfunction in hypertrophic cardiomyopathy. Circulation. 2021;144:1714‐1731. 34672721 10.1161/CIRCULATIONAHA.121.053575 PMC8608736

[32] Beger HG , Rau B , Mayer J , et al. Natural course of acute pancreatitis. World J Surg. 1997;21:130‐135. 8995067 10.1007/s002689900204

[33] Knottnerus SJG , Mengarelli I , Wüst RCI , et al. Electrophysiological abnormalities in VLCAD deficient hiPSC‐cardiomyocytes can be improved by lowering accumulation of fatty acid oxidation intermediates. Int J Mol Sci. 2020;21:2589. 32276429 10.3390/ijms21072589 PMC7177397

[34] Cabrero A , Alegret M , Sanchez RM , et al. Increased reactive oxygen species production down‐regulates peroxisome proliferator‐activated alpha pathway in C2C12 skeletal muscle cells. J Biol Chem. 2002;277:10100‐10107. 11792699 10.1074/jbc.M110321200

[35] Yao J , Miao Y , Zhang Y , et al. Dao‐Chi powder ameliorates pancreatitis‐induced intestinal and cardiac injuries via regulating the Nrf2‐HO‐1‐HMGB1 signaling pathway in rats. Front Pharmacol. 2022;13:922130. 35899121 10.3389/fphar.2022.922130 PMC9310041

[36] Wang Y , Chen M . Fentanyl ameliorates severe acute pancreatitis‐induced myocardial injury in rats by regulating NF‐κB signaling pathway. Med Sci Monit. 2017;23:3276‐3283. 28680032 10.12659/MSM.902245 PMC5510983

[37] Albasher G , Alkahtani S , Al‐Harbi LN . Urolithin A prevents streptozotocin‐induced diabetic cardiomyopathy in rats by activating SIRT1. Saudi J Biol Sci. 2022;29:1210‐1220. 35241966 10.1016/j.sjbs.2021.09.045 PMC8865018

[38] Luan P , D'Amico D , Andreux PA , et al. Urolithin A improves muscle function by inducing mitophagy in muscular dystrophy. Sci Transl Med. 2021;13:eabb0319. 33827972 10.1126/scitranslmed.abb0319

[39] Chistiakov DA , Shkurat TP , Melnichenko AA , et al. The role of mitochondrial dysfunction in cardiovascular disease: a brief review. Ann Med. 2018;50:121‐127. 29237304 10.1080/07853890.2017.1417631

[40] Ritterhoff J , Tian R . Metabolism in cardiomyopathy: every substrate matters. Cardiovasc Res. 2017;113:411‐421. 28395011 10.1093/cvr/cvx017 PMC5852620

[41] Bagby GJ , Spitzer JA . Lipoprotein lipase activity in rat heart and adipose tissue during endotoxic shock. Am J Physiol. 1980;238:H325‐330. 6989270 10.1152/ajpheart.1980.238.3.H325

[42] Divakaruni AS , Hsieh WY , Minarrieta L , et al. Etomoxir inhibits macrophage polarization by disrupting CoA homeostasis. Cell Metab. 2018;28:490‐503. 30043752 10.1016/j.cmet.2018.06.001 PMC6125190

[43] Feingold K , Kim MS , Shigenaga J , et al. Altered expression of nuclear hormone receptors and coactivators in mouse heart during the acute‐phase response. Am J Physiol Endocrinol Metab. 2004;286:E201‐207. 14701665 10.1152/ajpendo.00205.2003

[44] Drosatos K , Drosatos‐Tampakaki Z , Khan R , et al. Inhibition of c‐Jun‐N‐terminal kinase increases cardiac peroxisome proliferator‐activated receptor alpha expression and fatty acid oxidation and prevents lipopolysaccharide‐induced heart dysfunction. J Biol Chem. 2011;286:36331‐36339. 21873422 10.1074/jbc.M111.272146 PMC3196095

[45] Rossi MA , Celes MR , Prado CM , et al. Myocardial structural changes in long‐term human severe sepsis/septic shock may be responsible for cardiac dysfunction. Shock. 2007;27:10‐18. 17172974 10.1097/01.shk.0000235141.05528.47

[46] Wasyluk W , Nowicka‐Stążka P , Zwolak A . Heart metabolism in sepsis‐induced cardiomyopathy‐unusual metabolic dysfunction of the heart. Int J Environ Res Public Health. 2021;18:7598. 34300048 10.3390/ijerph18147598 PMC8303349

[47] Hunter WG , Kelly JP . Metabolomic profiling identifies novel circulating biomarkers of mitochondrial dysfunction differentially elevated in heart failure with preserved versus reduced ejection fraction: evidence for shared metabolic impairments in clinical heart failure. J Am Heart Assoc. 2016;5:e003190. 27473038 10.1161/JAHA.115.003190 PMC5015273

[48] Guo X , Hong T , Zhang S , et al. IL‐13 alleviates cardiomyocyte apoptosis by improving fatty acid oxidation in mitochondria. Front Cell Dev Biol. 2021;9:736603. 34604237 10.3389/fcell.2021.736603 PMC8484794

[49] Toney AM , Fan R , Xian Y , et al. Urolithin A, a gut metabolite, improves insulin sensitivity through augmentation of mitochondrial function and biogenesis. Obesity (Silver Spring). 2019;27:612‐620. 30768775 10.1002/oby.22404

[50] Tessier JP , Thurner B , Jüngling E , et al. Impairment of glucose metabolism in hearts from rats treated with endotoxin. Cardiovasc Res. 2003;60:119‐130. 14522413 10.1016/s0008-6363(03)00320-1

[51] Dhainaut JF , Huyghebaert MF , Monsallier JF , et al. Coronary hemodynamics and myocardial metabolism of lactate, free fatty acids, glucose, and ketones in patients with septic shock. Circulation. 1987;75:533‐541. 3815765 10.1161/01.cir.75.3.533

[52] Birkenfeld AL , Jordan J , Dworak M , et al. Myocardial metabolism in heart failure: purinergic signalling and other metabolic concepts. Pharmacol Ther. 2019;194:132‐144. 30149104 10.1016/j.pharmthera.2018.08.015

[53] Randle PJ , Garland PB , Hales CN , et al. The glucose fatty‐acid cycle. Its role in insulin sensitivity and the metabolic disturbances of diabetes mellitus. Lancet. 1963;1:785‐789. 13990765 10.1016/s0140-6736(63)91500-9

[54] Zheng Z , Ma H , Zhang X , et al. Enhanced glycolytic metabolism contributes to cardiac dysfunction in polymicrobial sepsis. J Infect Dis. 2017;215:1396‐1406. 28368517 10.1093/infdis/jix138 PMC5451607

[55] Hu Q , Yao J , Wu X , et al. Emodin attenuates severe acute pancreatitis‐associated acute lung injury by suppressing pancreatic exosome‐mediated alveolar macrophage activation. Acta Pharm Sin B. 2022;12:3986‐4003. 36213542 10.1016/j.apsb.2021.10.008 PMC9532455

[56] Hu Q , Zhang S , Yang Y , et al. Extracellular vesicle ITGAM and ITGB2 mediate severe acute pancreatitis‐related acute lung injury. ACS Nano. 2023;17:7562‐7575. 37022097 10.1021/acsnano.2c12722 PMC10134486

[57] Wang Y , Jasper H , Toan S , et al. Mitophagy coordinates the mitochondrial unfolded protein response to attenuate inflammation‐mediated myocardial injury. Redox Biol. 2021;45:102049. 34174558 10.1016/j.redox.2021.102049 PMC8246635

[58] Shriver LP , Manchester M . Inhibition of fatty acid metabolism ameliorates disease activity in an animal model of multiple sclerosis. Sci Rep. 2011;1:79. 22355598 10.1038/srep00079 PMC3216566

[59] Shen S , He F , Cheng C , et al. Uric acid aggravates myocardial ischemia‐reperfusion injury via ROS/NLRP3 pyroptosis pathway. Biomed Pharmacother. 2021;133:110990. 33232925 10.1016/j.biopha.2020.110990

[60] Liang D , Jin Y , Lin M , et al. Down‐regulation of Xist and Mir‐7a‐5p improves LPS‐induced myocardial injury. Int J Med Sci. 2020;17:2570‐2577. 33029099 10.7150/ijms.45408 PMC7532474

[61] Zhang Y , Zhang Y , Halemahebai G , et al. Urolithin A, a pomegranate metabolite, protects pancreatic β cells from apoptosis by activating autophagy. J Ethnopharmacol. 2021;272:113628. 33246115 10.1016/j.jep.2020.113628

[62] Singh H , Morita T , Suzuki Y , et al. High sensitivity, high surface area enzyme‐linked immunosorbent assay (ELISA). Biomed Mater Eng. 2015;26:115‐127. 26684884 10.3233/BME-151561

[63] Schmidt J , Rattner DW , Lewandrowski K , et al. A better model of acute pancreatitis for evaluating therapy. Ann Surg. 1992;215:44‐56. 1731649 10.1097/00000658-199201000-00007 PMC1242369

[64] De‐Pu Z , Li‐Sha G , Guang‐Yi C , et al. The cholinergic anti‐inflammatory pathway ameliorates acute viral myocarditis in mice by regulating CD4(+) T cell differentiation. Virulence. 2018;9:1364‐1376. 30176160 10.1080/21505594.2018.1482179 PMC6141146

[65] Lkhagva B , Kao YH , Lee TI , et al. Activation of Class I histone deacetylases contributes to mitochondrial dysfunction in cardiomyocytes with altered complex activities. Epigenetics. 2018;13:376‐385. 29613828 10.1080/15592294.2018.1460032 PMC6140820

