# Supplementary material for: Urolithin A protects severe acute pancreatitis‐associated acute cardiac injury by regulating mitochondrial fatty acid oxidative metabolism in cardiomyocytes
Source: MedComm (2020). 2023 Dec 19;4(6):e459. doi: 10.1002/mco2.459 (PMC10728757; doi:10.1002/mco2.459)
Supplement: Supplementary file 1 — Supporting Information [file MCO2-4-e459-s001.pdf]

Urolithin A protects severe acute pancreatitis-associated acute cardiac injury by regulating mitochondrial fatty acid oxidative metabolism in cardiomyocytes

Yue Yang[1], Qian Hu[1], Hongxin Kang[1], Juan Li[1], Xianlin Zhao[1], Lv Zhu[1], Wenfu Tang[1], Meihua Wan[1, 2, *]

[1]Department of Integrated Traditional Chinese and Western Medicine, West China Hospital of Sichuan University, Chengdu 610041, China
[2]West China Hospital (Airport) Hospital of Sichuan University, Chengdu 610299, China

*** Correspondence**
Meihua Wan, Department of Integrated Traditional Chinese and Western Medicine, National Clinical Research Center for Geriatrics, West China Hospital, Sichuan University, 610041, Chengdu, China; The First People's Hospital of Shuangliu District, Chengdu 610299, China
Email: wanmh@scu.edu.cn (Meihua Wan)

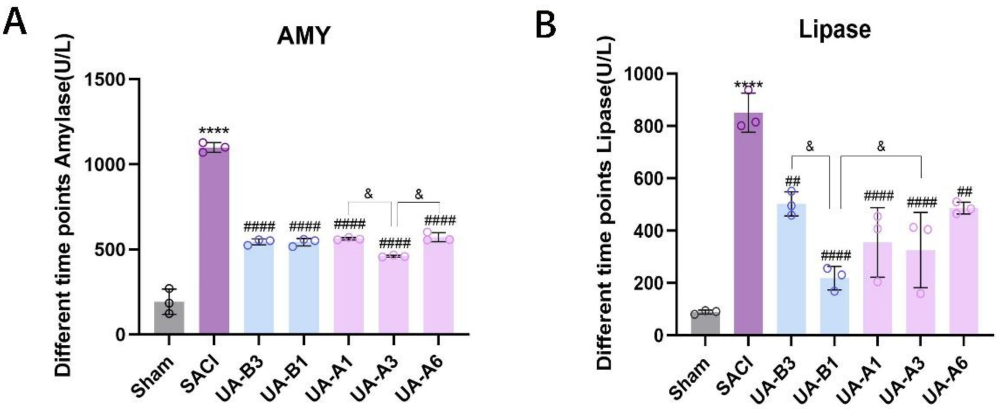

FIGURE S1 Exploration of the optimal timing of Urolithin A for SACI. (A-B) Serum amylase and lipase levels in the sham group and in the groups with different UA treatment time points (3 h before SACI induction, 1 h before SACI induction, 1 h after SACI induction, 3 h after SACI induction, 6 h after SACI induction) (n=3). The results are presented as the mean ± SD. ****p < 0.0001 vs. sham group；&p < 0.05 vs. UA-A3 or UA-B1 group；##p < 0.01, ####p < 0.0001 vs. SACI group, by one-way ANOVA tests followed by Tukey tests.

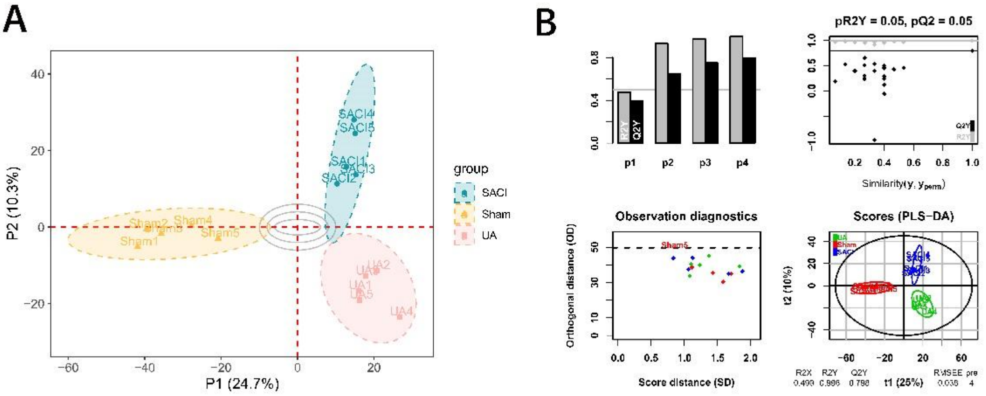

FIGURE S2 Untargeted metabolomics analysis. (A) PLS-DA model in the Sham, SACI, UA groups (n=5). (B) Validation of the PLS-DA model in the Sham, SACI, and UA groups (n=5).

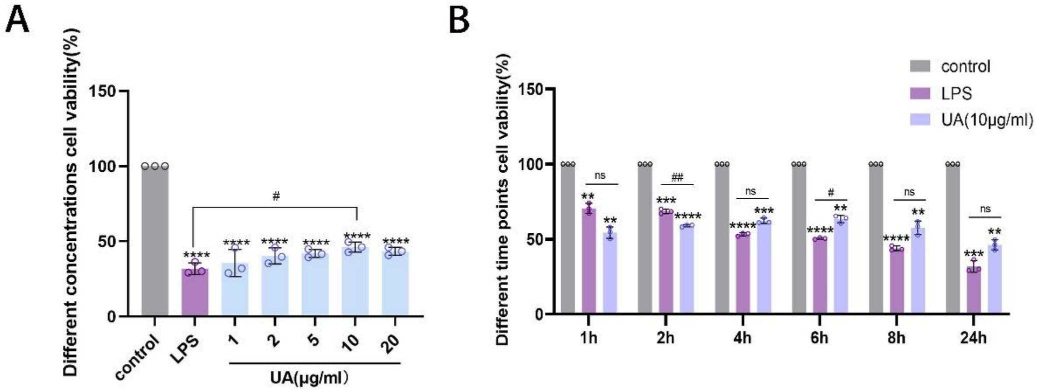

FIGURE S3 Detection of primary cardiomyocytes viability using CCK-8 assay. (A) Viability of LPS-induced injured cells after treatment by Urolithin A at different concentrations:1, 2, 5, 10 and 20 μg/mL for 24 h (n=3). (B) Viability of LPS-induced injured cells following treatment by Urolithin A (10 μg/ml) at different intervention intervals: 1, 2, 4, 6, 8 and 24 h (n=3). The results are presented as the mean ± SD. **p < 0.01, ***p < 0.005, ****p < 0.0001 vs. control group; #p < 0.05, ##p < 0.01 vs. LPS group, by one-way ANOVA tests followed by Tukey tests.

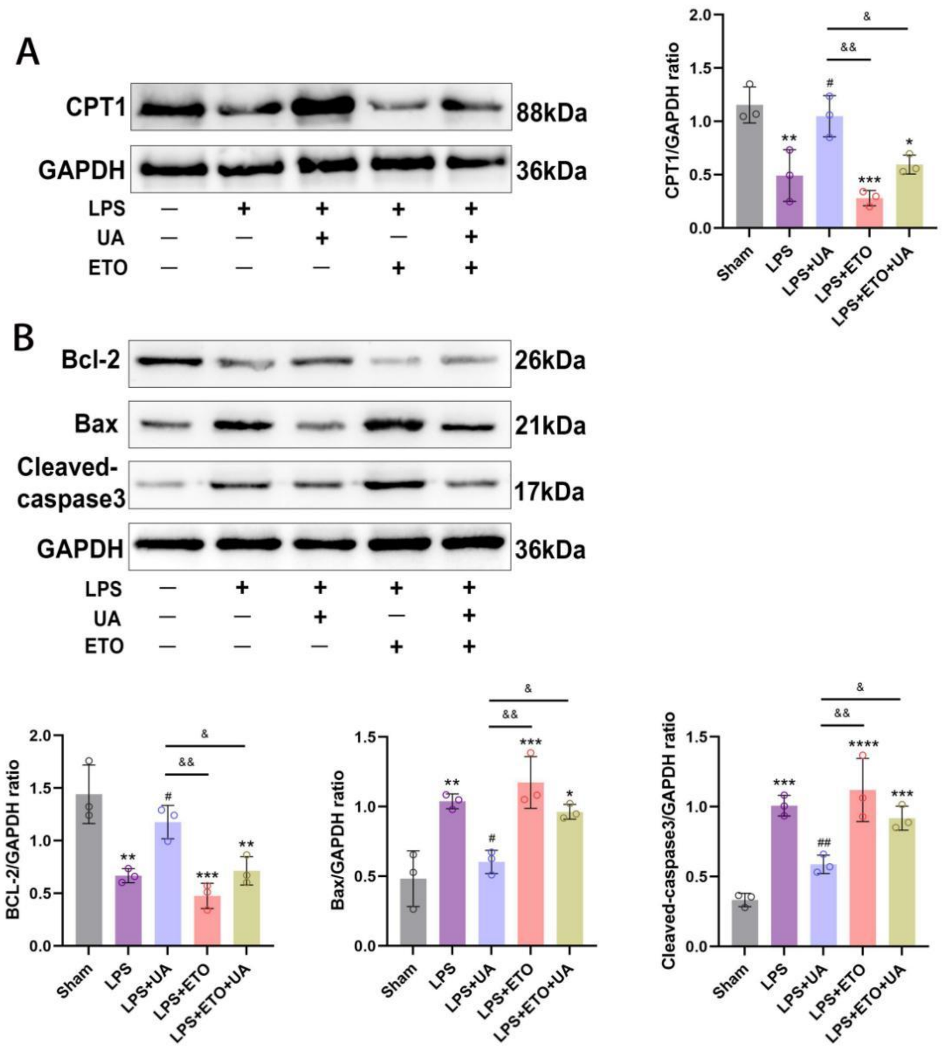

FIGURE S4 Urolithin A attenuates cardiomyocyte injury by regulating CPT1. (A) Western blotting and quantitative analyses of CPT1 in the sham group (sham), LPS-induced injured group (LPS), and LPS plus Urolithin A treatment group (LPS+UA), LPS plus etomoxir inhibitor group (LPS+ETO), LPS plus Urolithin A treatment and etomoxir inhibitor group (LPS+ETO+UA) (n=3). (B) Western blotting and quantitative analyses of Bcl-2, Bax, Cleaved-Caspase3 in each group (n=3). The results are presented as the mean ± SD. *P < 0.05, **P < 0.01, ***P < 0.005 vs. sham group; #p < 0.05, ##p < 0.01 vs. LPS group, &p < 0.05, &&p < 0.01 vs. LPS+UA group, by one-way ANOVA tests followed by Tukey tests.
